# 优化解决肺癌患者在新型冠状病毒肺炎疫情期间诊疗问题及心理问题的探索

**DOI:** 10.3779/j.issn.1009-3419.2020.101.18

**Published:** 2020-04-20

**Authors:** 海燕 徐, 科 杨, 广建 杨, 路 杨, 玉玲 米, 晓红 崔, 敏 杨, 丹 王, 燕 王

**Affiliations:** 1 100021 北京，国家癌症中心/国家肿瘤临床医学研究中心/中国医学科学院北京协和医学院肿瘤医院综合科 Department of Comprehensive Oncology, National Cancer Center/National Clinical Research Center for Cancer/Cancer Hospital, Chinese Academy of Medical Sciences and Peking Union Medical College, Beijing 100021, China; 2 100005 北京，北京市朝阳区桓兴肿瘤医院综合科 Department of Comprehensive Oncology, Huanxing Cancer Hospital, Chaoyang District, Beijing 100005, China; 3 100021 北京，国家癌症中心/国家肿瘤临床医学研究中心/中国医学科学院北京协和医学院肿瘤医院肿瘤内科 Department of Medical Oncology, National Cancer Center/National Clinical Research Center for Cancer/Cancer Hospital, Chinese Academy of Medical Sciences and Peking Union Medical College, Beijing 100021, China; 4 100122 北京，北京市朝阳区三环肿瘤医院 Lung Cancer Center, Sanhuan Cancer Hospital, Beijing 100122, China; 5 100088 北京，首都医科大学附属北京安定医院抑郁治疗中心 Depression Treatment Center, Beijing Anding Hospital, Capital Medical University, Beijing 100088, China

**Keywords:** 肺肿瘤, 新冠肺炎, 心理健康, Lung neoplasms, Novel coronavirus pneumonia, Mental health

## Abstract

**背景与目的:**

随着新冠肺炎（novel coronavirus pneumonia, NCP）在全世界范围内的迅速蔓延及防控工作的升级，肺癌患者常规诊疗需求因疫情原因受到不同程度的限制，有必要了解肺癌患者在疫情期间诊疗需求及心理健康情况，为后续的诊疗提供合理化建议。

**方法:**

采用电子问卷的形式，2020年3月4日7点发放，至3月6日7点截止，48 h收回368份问卷，覆盖了25个省自治区/直辖市。

**结果:**

368例肺癌患者，18例未行抗肿瘤治疗，最终纳入分析的患者共350例。229例口服靶向治疗，化疗或免疫治疗治疗121例。41.3%静脉注射化疗或免疫治疗患者出现治疗中断，中断比例较口服靶向治疗高（21.0%）。无论是口服靶向治疗还是静脉化疗或免疫治疗，超过60%患者出现影像学检查延迟。近1/3患者出现新发症状或原有症状加重，26.6%-28.9%患者通过网络咨询后更改了治疗方案。40%-75%肺癌患者在NCP期间存在心理健康问题，95%以上的肺癌患者对国家采取疫情防控措施表示支持和有信心。

**结论:**

在NCP期间，肺癌患者诊疗需求未得到满足，以化疗或免疫治疗的患者更明显，医疗机构恢复工作时应考虑优先解决。同时对患者存在的心理健康问题，应予以关注和疏解。

## Abstract (en)

**Background and objective:**

With the rapid spread of novel coronavirus pneumonia (NCP) worldwide and the escalation of prevention and control efforts, the routine medical needs of patients have been restricted. The aims were to investigate medical needs of lung cancer patients and their mental health status during the epidemic periods, so as to provide rational recommendations for subsequent diagnosis and treatment.

**Methods:**

The questionnaire was sent in the form of an electronic questionnaire at 7am on 4^th^, March, 2020, until 7am 6^th^, March, 2020, 368 questionnaires were recollected from 25 provinces (autonomous regions/municipalities) in 48 h.

**Results:**

Of the 368 patients, 18 patients were excluded as they didn't receive anti-tumor treatment, and 350 patients were included in the final analysis. 229 cases were treated with oral targeted drugs, and 121 cases were treated with chemotherapy or immunotherapy. 41.3% of patients treated with intravenous chemotherapy or immunotherapy experienced treatment discontinuation, and the proportion of treatment discontinuation in chemotherapy or immunotherapy was higher than those treated with oral targeted drugs (21.0%). Whether oral targeted drugs or intravenous chemotherapy or immunotherapy, more than 60% of patients experienced delays in imaging examinations. Nearly one third of patients developed new symptoms or exacerbation of existing symptoms. 26.6%-28.9% of patients have changed their treatment plans through online consultation. During novel coronavirus pneumonia, 40%-75% of lung cancer patients have mental health problems, and more than 95% of patients support government's prevention and control measures.

**Conclusions:**

During the emergence of NCP, the medical needs of patients with lung cancer have not been enough, especially those who discontinued chemotherapy or immunotherapy. When medical institution resumes work, priority should be given to them. At the same time, mental health problems of patients should be valued and resolved timely.

新型冠状病毒肺炎（novel coronavirus pneumonia, NCP）是一种由新型冠状病毒引发的急性呼吸道传染性疾病。其传播者为确诊患者或隐性感染者，主要的传播方式为呼吸道飞沫和密切接触传播^[1]^。另外，该病具有传播速度快且接触后发病率较高的特点，并且可以出现人际传播^[2, 3]^。因此，自2020年1月20日，国家卫生健康委员会决定将该病纳入法定传染病乙级管理，但采取甲类传染病的预防、控制措施。大多NCP以轻症为主，常表现为发热、咳嗽、乏力等症状，重症患者可快速出现严重低氧血症、多器官功能衰竭、甚至死亡，死亡率高达60%^[4]^。随着NCP在全世界范围内的迅速扩大及防控工作的升级，肺癌患者常规医疗行为因疫情原因受到不同程度的限制，也给从事肺癌诊疗的工作者在新的形式下提出新的挑战。有必要了解肺癌患者在疫情期间诊疗需求及心理健康情况，以便医疗工作者及医疗机构在疫情期间及时调整工作流程，做到防疫、防癌两不误，使肺癌患者平稳度过NCP的特殊时期。本研究采取电子问卷的形式进行调查，调查肺癌患者在疫情期间面临的就医问题以及心理健康情况，为肺癌患者后续的诊疗工作提出合理化建议。

## 1 对象与方法

### 1.1 研究对象

2020年3月4日-2020年3月6日期间，由中国医学科学院肿瘤医院设计并征求具有心理咨询资格的医生，设计了肺癌患者在NCP期间就医行为及心理健康的调查问卷APP。本问卷调查对象为NCP疫情发生之前在我院经组织学及病理学确诊的肺癌患者。

### 1.2 研究方法

调查工具：采用自制的电子问卷调查表，由本院指定的内科医生负责通过不同肺癌治疗的微信群发放调查问卷、并在APP里面填写了详细说明问卷填写的要求、注意事项，调查内容分为四个部分：①患者一般情况：包括年龄、性别、居住地、婚姻、文化程度、就诊医院、病理类型、目前治疗方式、分期情况以及生活状态等情况；②就医行为；③心理健康；④患者对国家和医院采取防治疫情的态度。该研究采取线上调查方式，包括在线填写的调研方法、匿名在线收集问卷以及实时数据传输。

### 1.3 统计学方法

该研究应用SPSS 16.0软件包进行统计分析。所有患者基线资料应用描述性统计进行分析，计数资料应用例数和百分比（%）来表示。应用Excel软件进行作图。

## 2 结果

### 2.1 调查对象的一般情况

从2020年3月4日早7点发出问卷，48 h后截止（2020年3月6日早7am），在线提取数据，共回收问卷368份（回收率100.0%），患者遍及自全国25个省、直辖市不同区域。男性169例，女性199例。77.2%来自北京以外区域，包括湖北地区8例，以腺癌为主（88.9%），其次为鳞癌（5.7%）和小细胞肺癌（5.4%）。参与调查的患者中目前的治疗以口服靶向治疗为主，占62.2%[其中包括表皮生长因子受体（epidermal growth factor receptor, EGFR）抑制剂或间变性淋巴瘤激酶（anaplastic lymphoma kinase, ALK）抑制剂为56.2%，小分子抗血管生成药物安罗替尼或阿帕替尼为6.0%]，次为化疗（22.3%），免疫或免疫联合化疗较少，占10.6%，还包括其他治疗4.9%（其中3.3%患者采用中药治疗和1.6%患者对症支持治疗）。患者的基本资料如表 1。

**1:** 368例患者基本资料[*n*（%）] Basic information of 368 patients [*n* (%)]

Variable	Data
Gender Male Female	169 (45.9) 199 (54.1)
Age（yr） < 40 40-60 > 60	36 (9.8) 188 (51.1) 144 (39.1)
Residence Beijing area Non-Beijing area Hubei area	84 (22.8) 284 (77.2) 8 (2.2)
Marital status Unmarried Married Widowed Divorced	10 (2.7) 341 (92.7) 12 (3.3) 5 (1.3)
Education level High school or low Bachelor level Master degree or above	205 (55.7) 147 (39.9) 16 (4.4)
Hospital grade Third-class Second-class Other hospital	344 (93.5) 16 (4.3) 8 (2.2)
Histological types Small cell Adenocarcinoma Squamous cell	20(5.4) 327(88.9) 21(5.7)
Treatment Oral targeted drug EGFR-TKI or ALK-TKI treatment Anlotinib or apatinib Chemotherapy Immunotherapy or immunotherapy plus chemotherapy Other	229 (62.2) 207 (56.2) 22 (6.0) 82 (22.3) 39 (10.6) 18 (4.9)
Clinical stage Locally advanced Metastatic	38 (10.3) 330 (89.7)
Self-care ability Complete self-care Needing help Without ability of self-care	327 (88.9) 32 (8.7) 9 (2.4)
Total	368 (100.0)
EGFR: epidermal growth factor receptor; TKI: tyrosine kinase inhibitors; ALK: anaplastic lymphoma kinase.	

### 2.2 肺癌患者NCP期间就医行为调查

368例肺癌患者中，18例患者未行抗肿瘤治疗，最终纳入分析的患者共350例。超过一半以上（59.4%）患者医疗需求未得到满足，表现为口服靶向治疗的中断、化疗或免疫中断、影像学检查延期。其中以化疗或免疫治疗患者较为突出（图 1A）。

**1:**
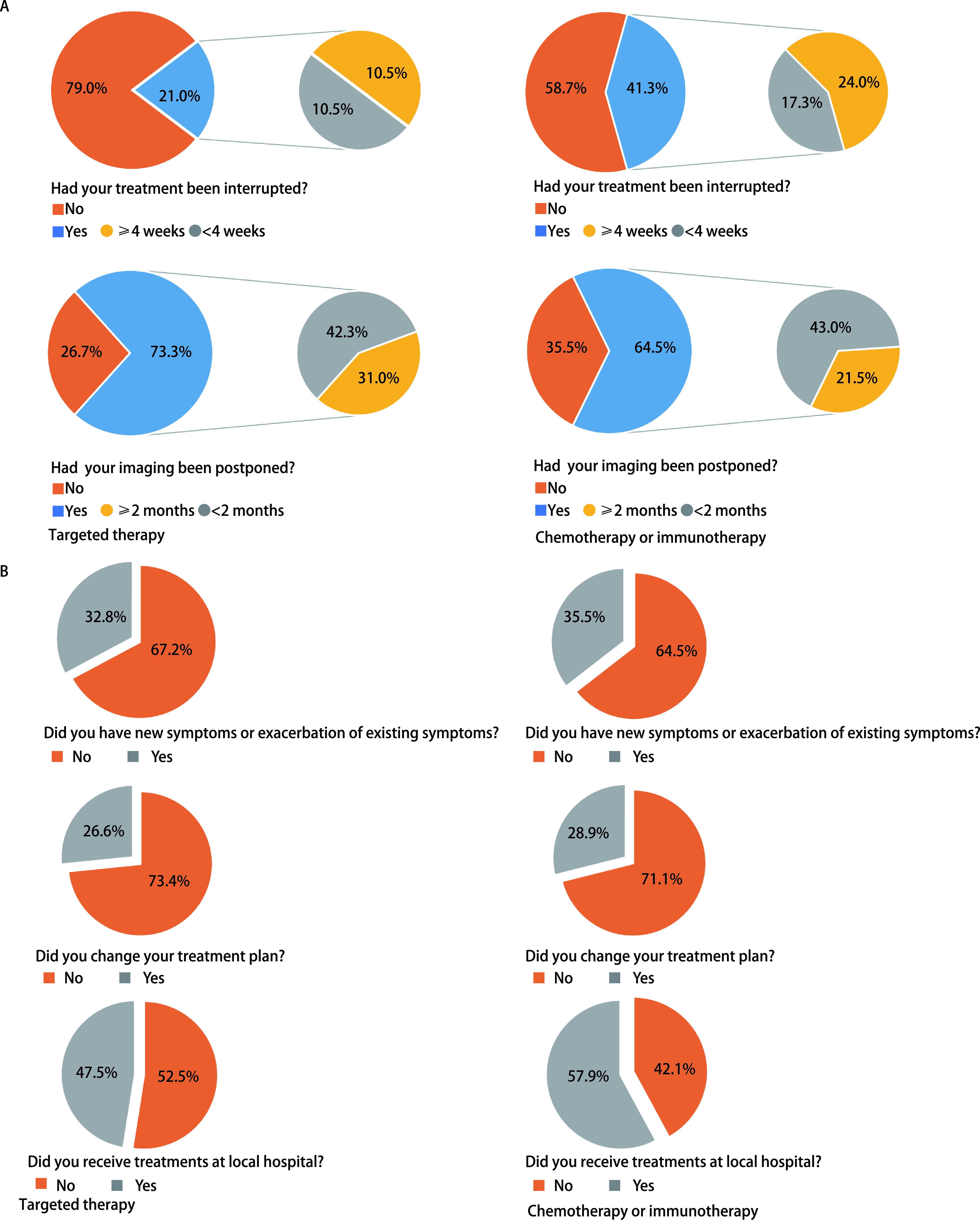
肺癌患者在NCP期间就医情况调查。A：肺癌患者治疗延迟情况；B：肺癌患者治疗改变的情况。 A survey of medical treatment for patients with lung cancer during novel coronavirus pneumonia. A: Delays in treatment of patients with lung cancer; B: Changes in treatment of patients with lung cancer. NCP: novel coronavirus pneumonia.

#### 2.2.1 肺癌患者在NCP期间治疗变化的调查

229例患者目前的治疗是口服靶向治疗，治疗中断比例较低，占21.0%，但73.3%患者出现影像学检查延迟，其中31.0%距上次影像学检查时间超过2个月。

121例患者目前的治疗方法是静脉注射化疗或免疫治疗，41.3%患者出现中断治疗，中断比例较口服靶向治疗高。64.5%患者影像学检查延期，其中21.5%距上次影像学检查时间超过2个月。

无论是口服靶向药物还是化疗或免疫治疗的患者中，出现新发症状或原有症状加重的比例均有将近1/3，因此改变治疗方案比例分别为26.6%和28.9%。但更多化疗或免疫治疗的患者（57.9%）倾向于在当地医院进行（图 1B）。

#### 2.2.2 肺癌患者在NCP期间网络就医行为调查

所有接受治疗患者，无论是口服靶向治疗患者还是化疗或免疫治疗患者均表示医疗需求远远未得到满足，比例接近60%（表 2）。靶向治疗的人群中选择了网络咨询不足50%，其中33.6%的患者通过1次-2次的网络咨询解决了问题。86.1%的患者认为网络咨询对自己的病情有帮助。疫情期间，有53.7%的患者会咨询非主管医师，但是当咨询意见不同时，91.3%的患者选择以主管医师意见为准。

**2:** 350例肺癌患者在NCP期间就医需求[*n*（%）] Medical needs of 350 patients with lung cancer during novel coronavirus pneumonia [*n* (%)]

Questions	Targeted drugs (*n*=229)	Immunotherapy or chemotherapy (*n*=121)
Had your medical needs been met? Yes No	91 (39.7) 138 (60.3)	51 (42.1) 70 (57.9)
Would you go to the hospital? Yes No	170 (74.2) 59 (25.8)	107 (88.4) 14 (11.6)
Had you been to the hospital? Yes No	139 (60.7) 90 (39.3)	91 (75.2) 30 (24.8)
Did you take part in the online consultation? Yes 1-2 times ≥3 times No	108 (47.2) 77 (33.6) 31 (13.6) 121 (52.8)	53 (43.8) 30 (24.8) 23 (19.0) 68 (56.2)
Was online consultation helpful?a Yes No	93 (86.1) 15 (13.9)	48 (90.6) 5 (9.4)
Could you contact your doctor in charge? Yes No	163 (71.2) 66 (28.8)	109 (90.0) 12 (10.0)
Which doctor did you consult? Doctor in-charge Other doctor	106 (46.3) 123 (53.7)	72 (59.5) 49 (40.5)
Who would you accept when the opinions were inconsistent after consultation? Doctor in-charge Network doctor Local doctor	209 (91.3) 16 (7.0) 4 (1.7)	114 (94.2) 6 (5.0) 1 (0.8)
Will you change the doctor in charge after NCP? Yes No	15 (6.6) 214 (93.4)	6 (5.0) 115 (95.0)
^a^: Totally 108 patients in the targeted drugs group and 53 cases in the immunotherapy or chemotherapy group took part in the online consultation and so only those patients participated in the investigation of this question.		

化疗或免疫治疗的患者网络咨询比例与靶向治疗相当（43.8% *vs* 47.2%），但19.0%通过进行了3次以上网络咨询，高于靶向治疗的13.6%。约60%化疗或免疫治疗患者咨询主管医生，当咨询意见不同时，94.2%的患者选择以主管医师意见为准。

### 2.3 肺癌患者在疫情期间心理健康情况

350例患者，316例处于居家隔离，34例因病情需要住院治疗，其中有3例患者因新冠住院治疗。从图 2可以看出40%-75%肺癌患者在NCP期间存在一定比例的紧张、睡眠障碍、空虚、易发脾气等情况，近一半患者出现沮丧情绪（图 2）。但94%患者对战胜NCP抱有很大信心。

**2:**
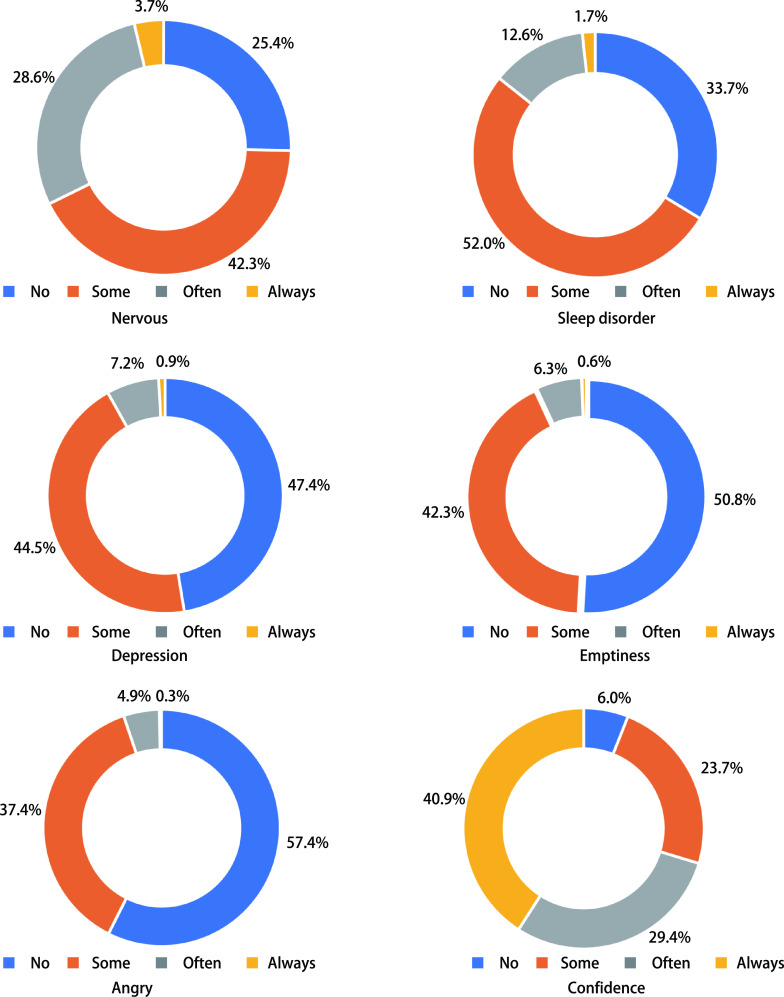
肺癌患者在NCP期间心理健康情况 Mental health status of patients with lung cancer during times of NCP

### 2.4 肺癌患者对国家和医院采取疫情防控措施的态度

350例肺癌患者，几乎所有患者（99.7%）表示出门会戴口罩、减少聚集性聚会。97.7%（342例）肺癌患者支持国家采取专门医院和专门科室集中收治NCP的患者。96.3%（337例）表示支持医院采取的三级预诊分诊措施，进行全方位筛查，96.9%（339例）表示支持科室分区诊疗工作，95.4%（334例）患者表示支持医生对患者采取单间输液措施，91.7%（321例）表示支持对于疑似患者进行定点医院进行筛查的态度，减少接触性感染（表 3）。

**3:** 350例患者对国家、医院在NCP期间所采取防控措施的态度[*n*（%）] 350 patients' attitudes to prevention and control measures taken by the government and hospital during novel coronavirus pneumonia [*n* (%)]

Questions	Favor	Anyway	Against
Wear masks when going out and reduce gathering activities	349 (99.7)	0 (0.0)	1 (0.3)
Patients of NCP need to be treated in a special hospital and wards	342 (97.7)	7 (2.0)	1 (0.3)
The hospital implements three-level pre-screening	337 (96.3)	8 (2.3)	5 (1.4)
Patients are divided into three areas: pre-diagnosis area, waiting area, and consultation area, and only one person allowed	339 (96.9)	8 (2.3)	3 (0.8)
Single room for patients who required intravenous infusion during NCP	334 (95.4)	14 (4.0)	2 (0.6)
Patients should go to a designated hospital for screening, when the cause of fever could not be identified	321 (91.7)	7 (2.0)	22 (6.3)

## 3 讨论

NCP作为一种新发的传染病，短期内蔓延到多个国家和地区，引发了全球的公共卫生事件^[5, 6]^。癌症患者比非癌症患者具有更高的严重事件发生风险，肺癌患者是新型冠状肺炎易感人群^[5, 7]^。本调查以已确诊正在治疗的肺癌患者作为调查对象，对这个群体具有一定代表性。在NCP疫情下，本研究调查新型冠状肺炎期间进行口服靶向药物及小分子抗血管药物治疗和静脉输注化疗或免疫治疗的肺癌患者医疗需求受到一定的限制，以静脉化疗或免疫治疗患者为著，部分出现治疗中断和影像学检查延迟的情况。41.3%化疗或免疫治疗的患者出现治疗中断，治疗中断比例较口服靶向药物治疗高（21.0%）。此外，影像学检查延期比例较高（64.5%），距上次影像学检查时间超过2个月比例达21.5%。近1/3的患者出现新发症状或原有症状加重，后与主管医生进行沟通，接近1/3的患者更改了治疗方案。近50%-60%患者选择在当地医院进行治疗，进一步提示医疗机构在逐渐恢复工作时以最先解决这一部分患者的医疗需求为主要任务。我们还分析了71例未中断化疗和免疫治疗的患者，67例患者生活能够自理，4例患者需要部分帮助，绝大多数患者年龄在60岁以下，在住院前影像学排查NCP的前提下，进行日间化疗，一人一间，一位家属陪同，患者及家属全程戴好口罩，配合医院在指定的区域进行治疗，治疗结束后密切进行随访，进行不良反应的教育。90%的进行化疗或免疫治疗的患者与主管医生建立良好的沟通渠道。进一步提示在适度放开的阶段，化疗或免疫治疗的患者在排除NCP的前提下可逐步、适度开展医疗工作，由于受到客观条件限制，在对合并症或严重不良反应的处理上不能像正常条件下那么到位，建议前期优先收治年轻、一般状态好、生活完全或部分自理、依从性高的患者，待各部门条件完善后再恢复所有的诊治。

在NCP的疫情期间，各大医院均推行线上会诊的治疗方式，对于选择线上网络咨询的患者而言，超过80%靶向治疗的患者认为网络咨询对自己有帮助，通过1次-2次网络咨询解决了问题，主管医师的意见仍然是患者最信赖的治疗选择。化疗与免疫治疗患者对网络咨询的依赖性更高，需要超过3次以上的网络咨询才能解决问题的比例较高，这一现象体现了慢病患者的长期、稳定、互信医患关系。但是，仍有超过一半的患者没有进行线上咨询，考虑可能的原因有病情稳定、普及程度不足等原因。鉴于疫情下，各个医院均会受到不同程度的影响，线下就诊方式受到极大的冲击。线上就诊方式和医院之间的合作显得尤为重要。结合此次疫情下，肺癌患者就诊方式的统计结果，可见临时推广或加强线上就诊，三甲医院可以和当地医院整合资源，优化合作体系和流程，解决部分患者就诊需求，也是执行国家分级诊疗的一种措施。衡敬之等^[8]^认为“互联网+”分级诊疗模式可以通过互联网实现医疗信息的双向乃至多向流动，让患者在不同级别的医院得到科学分流，缩短时空差距，有利于灵活调配医疗资源。此外，还可以将各级医疗机构、医疗活动参与者、管理者及社会相关机构有机结合，借助大数据使得各个环节连成一体，形成一个系统、灵动、协调的分级诊疗体系。张红文等^[9]^阐述了在大数据时代“互联网+”精准医疗健康发展的创新组织形式，建立医疗整合资源，各个医院间实现远程会诊，实现区域化合作。因此，在医疗资源不平衡及传染病来临时，医疗需求受到限制的情况，推广新的互联网医疗模式，加强不同等级医院间学科建设是我国医疗行业进一步发展的方向。

本调查显示多数肺癌患者对于NCP的防控很有信心，但是40%-75%患者存在紧张、焦虑、睡眠障碍、易发脾气、易激惹等情况，同时伴有沮丧心理变化。因此，患者的心理变化需要得到更多关注，尤其是刚刚恢复诊疗时，面对一线医务人员，患者憋在心中的不满和委屈会倾泄而出，而医务人员也无法满足患者所有要求，并解决他们所有的问题，这时候就很容易产生医患矛盾，所以提示医务人员在接诊时给予更多的耐心、细心和关心，工作中予以及时疏导，调整患者的紧张、焦虑情绪，尽可能避免矛盾和纠纷。在这种形势下，国家新型冠状病毒肺炎的联防联控工作机制印发了《新型冠状病毒感染的肺炎疫情紧急心理危机干预指导原则》，在各大互联网平台也为患者及家属提供了心理危机援助。王文婧等^[10]^发现传染病患者在临床治疗过程中应用心理干预，能够改善其心理状态及负性情绪。心理干预对个体心理健康具有积极的促进作用。

从肺癌患者对医院、国家采取疫情防控措施的态度来看，几乎所有患者支持外出一定带口罩、减少聚集性聚会的措施，97.8%受访者一致认为所有确诊或疑似患者由专门医院和专门病房来进行收治，疑似NCP的肺癌患者，先去定点医院进行筛查。在此期间，绝大多数肺癌患者支持医院采取三级预诊分诊制度，医院采取单向通道出入，医院门口是第一道关卡，就诊科室是第二道关卡，坐诊医生是第三道关卡，所有关卡都要详细询问流行病史、呼吸道症状及监测体温，做到早发现、早诊治。就诊时分为三个区域，包括等候区、候诊区、诊疗区，每次就诊一人，人员间隔1 m距离，减少接触性的感染机会。确实需要进行输液治疗的患者，按照医护人员安排的指定路线、指定位置进行输液治疗，遵循医院的规章制度，做到减少交叉性感染的机会。

综上所述，基于对肺癌患者的问卷调查，让我们了解到在NCP期间，确实存在肺癌患者就医需求不能满足的问题，其中，面临治疗中断是需要化疗和免疫治疗人群亟待解决的问题。同时肺癌患者在疫情期间存在一定比例的紧张、焦虑、易发脾气及易激惹等心理变化，容易在就诊时爆发，造成医患纠纷等问题也不能忽视。因此希望通过对患者产生问题的了解，给后续逐步恢复的医疗工作一点建议，争取更快、更精确地帮助患者解决问题。此外，网络咨询作为一种新模式，在疫情期间解决了一部分患者的就医问题，预示这种模式有很强生命力，估计在疫情过后也会有很大发展空间。疫情虽然打乱了常规，但也暴露了问题，让我们有机会反思和改善现有的不足，希望通过国家、医院、患者三方共同努力，尽快恢复安全、有序、平稳的医疗工作。

## References

[1] Special Expert Group for Control of the Epidemic of Novel Coronavirus Pneumonia of the Chinese Preventive Medicine Association (2020). An update on the epidemiological characteristics of novel coronavirus pneumonia (COVID-19). Zhonghua Liu Xing Bing Xue Za Zhi.

[2] Chan JF, Yuan S, Kok KH (2020). A familial cluster of pneumonia associated with the 2019 novel coronavirus indicating person-to-person transmission: a study of a family cluster. Lancet.

[3] Phelan AL, Katz R, Gostin LO (2020). The novel coronavirus originating in Wuhan, China: Challenges for global health governance. JAMA.

[4] Yang X, Yu Y, Xu J (2020). Clinical course and outcomes of critically ill patients with SARS-CoV-2 pneumonia in Wuhan, China: a single-centered, retrospective, observational study. Lancet Respir.

[5] Epidemiology Working Group for NCIP Epidemic Response, Chinese Center for Disease Control and Prevention (2020). The epidemiological characteristics of an outbreak of 2019 novel coronavirus disease (COVID-19) in China. Zhonghua Liu Xing Bing Xue Za Zhi.

[6] Wang C, Horby PW, Hayden FG (2020). A novel coronavirus outbreak of global health concern. Lancet.

[7] Liang W, Guan W, Chen R (2020). Cancer patients in SARS-CoV-2 infection: a nationwide analysis in China. Lancet Oncol.

[8] Heng JZ, Xu ZD (2019). New development of hierarchical diagnosis and treatment mode in the context of "Internet +" era. Xian Dai Yi Yuan Guan Li.

[9] Zhang HW, Cai YQ, Wang WJ (2019). Trends on the development of precision medical care. Zhongguo Yi Yuan.

[10] Wang WJ (2017). Effect of psychological nursing intervention on negative emotion in patients with infectious disease. Shenzhen Zhong Xi Yi Jie He Za Zhi.

